# Effect of Different Bone Grafting Materials and Mesenchymal Stem Cells on Bone Regeneration: A Micro-Computed Tomography and Histomorphometric Study in a Rabbit Calvarial Defect Model

**DOI:** 10.3390/ijms22158101

**Published:** 2021-07-28

**Authors:** Shiau-Ting Shiu, Wei-Fang Lee, Sheng-Min Chen, Liu-Ting Hao, Yuan-Ting Hung, Pin-Chuang Lai, Sheng-Wei Feng

**Affiliations:** 1 School of Dentistry, College of Oral Medicine, Taipei Medical University, Taipei 11031, Taiwan; 14473@s.tmu.edu.tw (S.-T.S.); b202105027@tmu.edu.tw (S.-M.C.); howardliu1022@gmail.com (L.-T.H.); jeff890805@gmail.com (Y.-T.H.); 2 Department of Dentistry, Shuang Ho Hospital, Taipei Medical University, New Taipei City 23561, Taiwan; 3 School of Dental Technology, College of Oral Medicine, Taipei Medical University, Taipei 11031, Taiwan; weiwei@tmu.edu.tw; 4 Department of Diagnosis and Oral Health, School of Dentistry, University of Louisville, Louisville, KY 40202, USA; ULDA550@louisville.edu; 5 Department of Dentistry, Division of Prosthodontics, Taipei Medical University Hospital, Taipei 11031, Taiwan

**Keywords:** MBCP, Bio-Oss, mesenchymal stem cell, dental pulp-derived mesenchymal stem cells, bone marrow-derived mesenchymal stem cells, bone regeneration

## Abstract

This study evaluated the new bone formation potential of micro–macro biphasic calcium phosphate (MBCP) and Bio-Oss grafting materials with and without dental pulp-derived mesenchymal stem cells (DPSCs) and bone marrow-derived mesenchymal stem cells (BMSCs) in a rabbit calvarial bone defect model. The surface structure of the grafting materials was evaluated using a scanning electron microscope (SEM). The multipotent differentiation characteristics of the DPSCs and BMSCs were assessed. Four circular bone defects were created in the calvarium of 24 rabbits and randomly allocated to eight experimental groups: empty control, MBCP, MBCP+DPSCs, MBCP+BMSCs, Bio-Oss+DPSCs, Bio-Oss+BMSCs, and autogenous bone. A three-dimensional analysis of the new bone formation was performed using micro-computed tomography (micro-CT) and a histological study after 2, 4, and 8 weeks of healing. Homogenously porous structures were observed in both grafting materials. The BMSCs revealed higher osteogenic differentiation capacities, whereas the DPSCs exhibited higher colony-forming units. The micro-CT and histological analysis findings for the new bone formation were consistent. In general, the empty control showed the lowest bone regeneration capacity throughout the experimental period. By contrast, the percentage of new bone formation was the highest in the autogenous bone group after 2 (39.4% ± 4.7%) and 4 weeks (49.7% ± 1.5%) of healing (*p* < 0.05). MBCP and Bio-Oss could provide osteoconductive support and prevent the collapse of the defect space for new bone formation. In addition, more osteoblastic cells lining the surface of the newly formed bone and bone grafting materials were observed after incorporating the DPSCs and BMSCs. After 8 weeks of healing, the autogenous bone group (54.9% ± 6.1%) showed a higher percentage of new bone formation than the empty control (35.3% ± 0.5%), MBCP (38.3% ± 6.0%), MBCP+DPSC (39.8% ± 5.7%), Bio-Oss (41.3% ± 3.5%), and Bio-Oss+DPSC (42.1% ± 2.7%) groups. Nevertheless, the percentage of new bone formation did not significantly differ between the MBCP+BMSC (47.2% ± 8.3%) and Bio-Oss+BMSC (51.2% ± 9.9%) groups and the autogenous bone group. Our study results demonstrated that autogenous bone is the gold standard. Both the DPSCs and BMSCs enhanced the osteoconductive capacities of MBCP and Bio-Oss. In addition, the efficiency of the BMSCs combined with MBCP and Bio-Oss was comparable to that of the autogenous bone after 8 weeks of healing. These findings provide effective strategies for the improvement of biomaterials and MSC-based bone tissue regeneration.

## 1. Introduction

Tissue engineering involves the application of biological and engineering principles for the repair and functional enhancement of human tissues [1]. In particular, for the reconstruction of craniofacial bone defects, interdisciplinary methods and concepts, including the use of suitable scaffold materials, feasible seed cells, secretome, and bioactive factors, are considered to be vital in the field of bone regeneration [2,3,4,5,6,7]. Although the autogenous bone is considered the gold standard for the reconstruction of bone defects, several disadvantages limit its clinical application, including the morbidity of the potential donor site, the requirement of additional surgery, and the low availability of a suitable autologous material [8,9].

Currently, synthetic bone grafting materials and xenografts are alternative choices that are gradually being increasingly used for reconstructing bone defects in clinical practice because of their biocompatibility and osteoconductive properties without the concerns of immune responses and disease transmission [10,11,12]. Synthetic bone grafting materials and xenografts can provide a scaffold for cell attachment, interaction, migration, and proliferation, and they provide structural support to the newly formed bone [12]. In addition, most bone graft materials are expected to be reabsorbed and replaced as the natural bone heals over a few months. However, the insufficient osteogenic ability, low osteoinductive property, and inadequate bone regeneration potential of synthetic bone grafting materials and xenografts reduce their capability in enhancing bone healing in large bone defects [1,10,13]. On the other hand, a combination of synthetic and xenograft bone grafting materials (Smartbone^®^) has been proposed and developed. Smartbone^®^ is a biohybrid bone substitute constituted of a bovine bone-derived matrix and a thin poly(L-lactide- co-ε-caprolactone) (PLCL) film integrated with RGD-containing collagen fragments, which can enhance the cell viability, cell adhesion, and biocompatibility [7].Therefore, stem cell-based therapy has emerged as an alternative strategy in bone tissue engineering to overcome the limitations of synthetic bone grafting materials and xenografts.

Mesenchymal stem cells (MSCs) are characterized by their self-renewal, multi-lineage differentiation, and immunomodulatory capacities in adult tissues [3,14,15]. MSCs can be isolated from numerous tissues, including the bone marrow, adipose tissue, dental pulp, peripheral blood, umbilical cord, and skeletal muscle [16]. Bone marrow-derived MSCs (BMSCs) are the most commonly used source of MSCs and possess more potent osteogenic and chondrogenic differentiation abilities [17,18]. In addition, BMSCs can effectively promote bone repair by secreting factors that stimulate endogenous repair processes or by directly contributing to new bone formation through differentiation into osteoblast-like cells [15,19]. Moreover, some studies have indicated that BMSCs can regulate inflammation, ameliorate tissue deterioration, and promote neovascularization, thereby facilitating the growth of new tissues [19,20].

DPSCs, which are dental pulp-derived MSCs, have been proposed as a potential cell source for bone tissue engineering because of their high feasibility, easy access, and noninvasive harvesting without ethical concerns [21,22]. DPSCs have exhibited strong angiogenic and osteogenic potential in vitro and in vivo with the ability to directly differentiate into or interact with endothelial cells and osteoblasts [18,23,24,25,26,27]. In addition, the DPSCs demonstrated a strong potential for proliferation and differentiation, along with paracrine properties; the in vivo implantation of DPSCs promoted bone regeneration and repaired bone defects [22,28,29,30]. DPSCs appear to be a promising cell source of MSCs for craniofacial bone regeneration because of their similar embryonic origins [30,31].

The efficacy of MSC-based therapy may be affected by the characteristics of bone grafting materials [11,32]. Previous in vivo studies have demonstrated that collagen scaffolds enriched with periodontal ligament-derived MSCs (PDLSCs) or MSC-derived secretome [4] and a xenohybrid bone graft (Smartbone^®^) combined with adipose-derived stromal vascular fraction or lyoseretome can effectively induce new bone formation, stimulate the recruitment of endogenous bone marrow MSCs, and promote an osteoinductive ability [5,7].

Moreover, selecting the most appropriate support scaffold and determining the optimal source of MSCs before implantation are mandatory for in vivo bone regeneration [32,33]. Therefore, we investigated the interaction between different types of bone grafting materials and different types of adult stem cells to determine the most favorable strategy for future clinical applications. The present study comprehensively evaluated the bone healing capacities of synthetic bone grafting materials (micro–macro biphasic calcium phosphate (MBCP)) and xenografts (Bio-Oss) with and without DPSCs and BMSCs compared with an empty defect and the autogenous bone in a rabbit calvarial bone defect model.

## 2. Results

### 2.1. Characterization of MBCP and Bio-Oss

**Figure 1:**
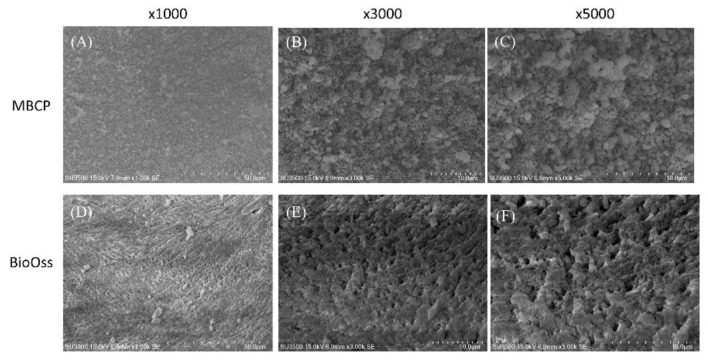
SEM images of MBCP and Bio-Oss at different magnifications (×1000, ×3000, and ×5000). (**A**–**C**) Morphology evaluation of MBCP at increasing magnification. (**D**–**F**) Morphology evaluation of Bio-Oss at increasing magnification.

As shown in Figure 1, the surface structures in both porous MBPC and Bio-Oss were homogeneous and had different patterns of macropore or micropore dimensions, with varying distributions and amounts. The two bone graft materials had beaded structurea. MBCP had a crystalline structure with a moderately rough surface (Figure 1A–C). In addition, Bio-Oss showed more porosity than MBCP. The surface structure of Bio-Oss exhibited clearly demarcated grain boundaries and a microporous layer (Figure 1D–F).

### 2.2. Characterization of DPSCs and BMSCs

**Figure 2:**
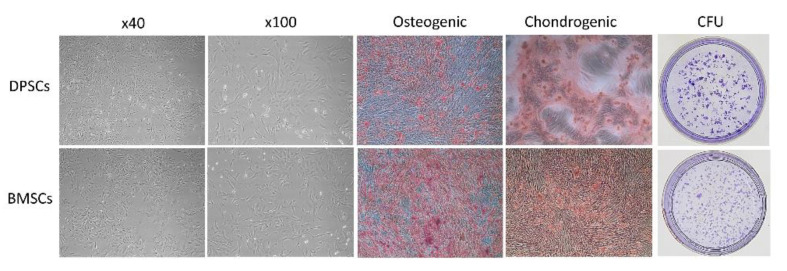
Characteristics of both the DPSCs and BMSCs.

Both DPSCs and BMSCs presented typical fibroblast-like and spindle-shaped morphologies, respectively, with homogeneous shapes and sizes (Figure 2). Regarding the multipotent differentiation potential, BMSCs had more osteogenic expression and calcium deposition staining than DPSCs; however, the chondrogenic differentiation capacity was similar between the BMSCs and DPSCs. Nevertheless, the number of CFUs of the DPSCs was higher than that of the BMSCs.

### 2.3. Micro-CT Measurements

**Figure 3:**
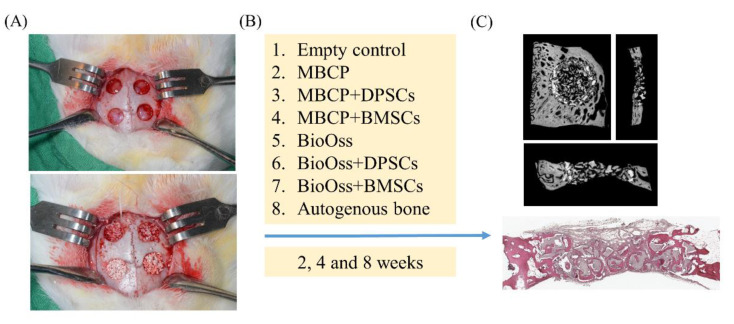
Schematic representation of the in vivo experiments was demonstrated. (**A**) A clinical photograph of the surgery and implantation of bone grafting materials for bone regeneration. Four circular bone defects measuring 6 mm in diameter were created using a trephine drill in surgical areas. (**B**) The bone defects were randomly allocated to eight experimental groups: empty control, MBCP, MBCP+DPSCs, MBCP+BMSCs, Bio-Oss, Bio-Oss+DPSCs, Bio-Oss+BMSCs, and autogenous bone. (**C**) Micro-CT and histological analysis.

The osteogenic potential of bone grafting materials and MSCs for bone defect repair was investigated using a rabbit calvarial defect model (Figure 3). A total of 30 mg of MBCP and Bio-Oss bone grafting materials containing 1 × 106 DPSCs or BMSCs were implanted into 6-mm-diameter defects created using a trephine drill. After 2, 4, and 8 weeks of healing, all bone blocks were collected for micro-CT, histological, and histomorphometric assessments (Figure 3).

**Figure 4:**
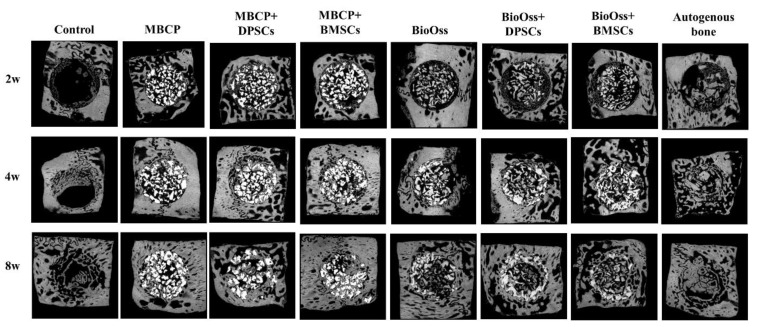
Micro-CT images of calvarial bone regeneration in the horizontal plane after 2, 4, and 8 weeks of healing.

As shown in Figure 4, the micro-CT data revealed that the autogenous bone group showed more favorable growth than the other experimental groups during the whole healing period. In the control group, few newly formed bones were observed only around the border of the defects after 2 weeks of healing. At 4 and 8 weeks, new bone formation gradually increased from the border to the center area. In the MBCP and Bio-Oss groups, new bone formation was observed that gradually increased not only around the border of the defects but also in the central area of the defects from 2 to 8 weeks (Figure 4). After the incorporation of undifferentiated DPSCs or BMSCs, the rate of new bone formation and the number of bony bridges markedly increased compared with those in the control or scaffold-only groups. Moreover, from 2 to 8 weeks, the rate of new bone formation increased, and more bone grafting materials were gradually resorbed.

**Figure 5:**
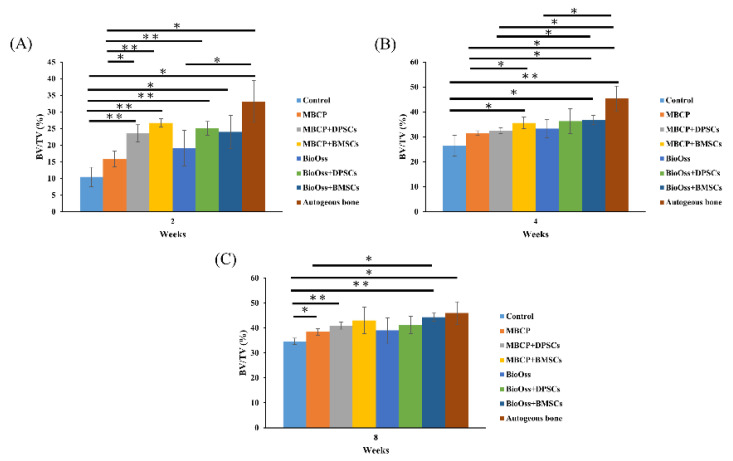
Statistical results of the bone volume/tissue volume (BV/TV) values after 2, 4, and 8 weeks of healing (* *p* < 0.05 and ** *p* < 0.01). (**A**) The BV/TV values in the autogenous bone group are significantly higher than other groups after 2 weeks of healing. (**B**) The BV/TV values of the MBCP+BMSCs, Bio-Oss+BMSCs and autogenous bone groups were no significantly different after 4 weeks of healing. (**C**) Similar BV/TV values were demonstrated among MBCP+DPSCs, MBCP+BMSCs, Bio-Oss+DPSCs, Bio-Oss+BMSCs and autogenous bone groups after 8 weeks of healing.

As shown in Figure 5A, the BV/TV value in the autogenous bone group was 33.1% ± 6.2%, which was significantly higher than those in the control (10.4% ± 2.9%), MBCP (15.9% ± 2.3%), and Bio-Oss (19.0% ± 5.3%) groups (*p* < 0.05) after 2 weeks of healing. However, comparable results were found in the experimental groups after the incorporation of DPSCs or BMSCs in MBCP and Bio-Oss. No significant differences in the BV/TV values were observed among the MBCP+DPSC (23.5% ± 2.6%), MBCP+BMSCs (26.8% ± 1.2%), Bio-Oss+DPSCs (25.1% ± 2.0%), and Bio-Oss+BMSCs (24.0% ± 4.9%) groups. In addition, the MBCP+DPSC and MBCP+BMSC groups exhibited higher BV/TV values than the MBCP-only group (*p* < 0.05), whereas the Bio-Oss, Bio-Oss+DPSC, and Bio-Oss+BMSC groups presented no significant differences (Figure 5A).

After 4 weeks of healing (Figure 5B), the BV/TV values of the MBCP+BMSC (35.7% ± 2.3%), Bio-Oss+BMSCs (36.9% ± 1.8%), and autogenous bone (45.4% ± 4.9%) groups were significantly higher than that of the empty control group (26.5% ± 4.1%; *p* < 0.05). The MBCP+BMSC group (35.66% ± 2.3%) exhibited a higher BV/TV value than the MBCP group (31.55% ± 0.9%); no significant difference in the BV/TV value was observed between the MBCP+BMSC (35.66% ± 2.3%) and MBCP+DPSC (32.4% ± 1.2%) groups. Furthermore, no differences were found among the Bio-Oss (33.3% ± 1.7%), Bio-Oss+DPSC (36.2% ± 5.0%), and Bio-Oss+BMSC (36.9% ± 1.7%) groups. After 8 weeks of healing (Figure 5C), the BV/TV values of the MBCP (38.4% ± 1.3%), MBCP+DPSC (41.0% ± 1.4%), Bio-Oss+BMSC (44.4% ± 1.7%), and autogenous bone (46.4% ± 4.5%) groups were significantly higher than that of the empty control group (34.6% ± 1.3%; *p* < 0.05). In addition, the Bio-Oss+BMSC group exhibited a higher BV/TV value than the MBCP group. Moreover, the MBCP+DPSC (41.0% ± 1.4%), MBCP+BMSC (43.0% ± 5.3%), Bio-Oss (39.0% ± 5.1%), Bio-Oss+DPSC (41.2% ± 3.4%), and Bio-Oss+BMSC (44.4% ± 1.7%) groups showed comparable capacities in new bone formation. These results indicated that bone grafting materials combined with DPSCs and BMSCs could promote new bone formation in calvarial bone defects.

### 2.4. Histological Observations

**Figure 6:**
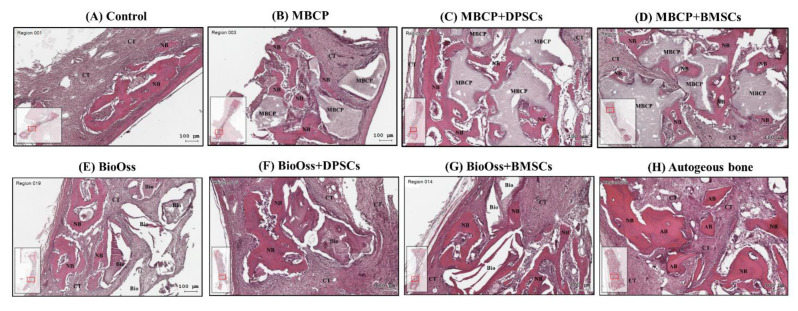
Histological analysis of calvarial bone defects after 2 weeks of healing in (**A**) the empty control, (**B**) MBCP, (**C**) MBCP+DPSCs, (**D**) MBCP+BMSCs, (**E**) Bio-Oss, (**F**) Bio-Oss+DPSCs, (**G**) Bio-Oss+BMSCs, and (**H**) autogenous bone groups. NB = new bone, CT = connective tissue, MBCP = MBCP, Bio = Bio-Oss, and AB = autogenous bone. Scale bar = 100 μm.

**Figure 7:**
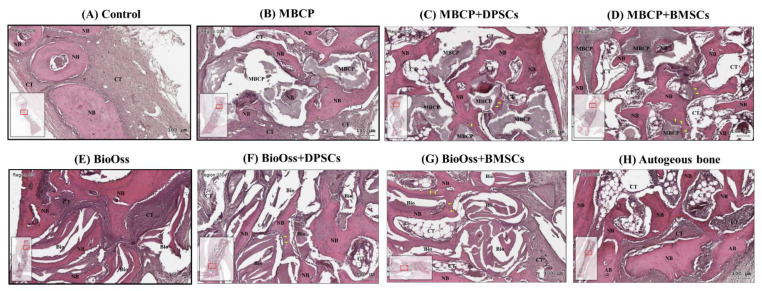
Histological analysis of calvarial bone defects after 4 weeks of healing in (**A**) the empty control, (**B**) MBCP, (**C**) MBCP+DPSCs, (**D**) MBCP+BMSCs, (**E**) Bio-Oss, (**F**) Bio-Oss+DPSCs, (**G**) Bio-Oss+BMSCs, and (**H**) autogenous bone groups. NB = new bone, CT = connective tissue, MBCP = MBCP, Bio = Bio-Oss, and AB = autogenous bone. Scale bar = 100 μm.

**Figure 8:**
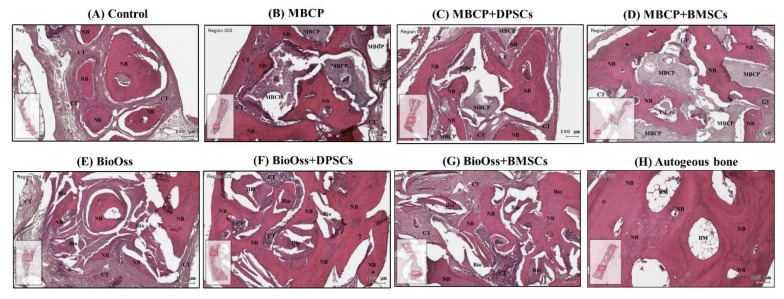
Histological analysis of calvarial bone defects after 8 weeks of healing in (**A**) the empty control, (**B**) MBCP, (**C**) MBCP+DPSCs, (**D**) MBCP+BMSCs, (**E**) Bio-Oss, (**F**) Bio-Oss+DPSCs, (**G**) Bio-Oss+BMSCs, and (**H**) autogenous bone groups. NB = new bone, CT = connective tissue, MBCP = MBCP, Bio = Bio-Oss, AB = autogenous bone, and BM = bone marrow. Scale bar = 100 μm.

In all the bone defect specimens, no signs of inflammation, infection, and necrosis induced by bone grafting materials or MSCs were observed. The histological findings of the bone defect samples with new bone formation and surrounding tissues for the eight experimental groups are shown in Figure 6, Figure 7 and Figure 8.

**Figure 9:**
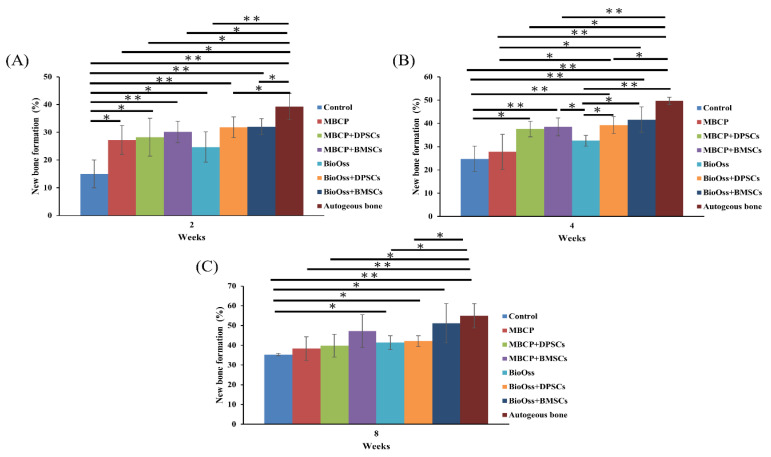
Statistical results of the histomorphometric analysis after 2, 4, and 8 weeks of healing (*p < 0.05 and **p < 0.01). (**A**) The new bone formation percentages in the autogenous bone group are significantly higher than other groups after 2 weeks of healing. (**B**) The new bone formation percentages of the Bio-Oss+BMSCs and autogenous bone groups were no significantly different after 4 weeks of healing. (**C**) Similar new bone formation percentages were demonstrated among MBCP+BMSCs, Bio-Oss+BMSCs and autogenous bone groups after 8 weeks of healing.

Overall, the histological and histomorphometric results indicated a higher new bone formation capacity in the MBCP+DPSC, MBCP+BMSC, Bio-Oss+DPSC, Bio-Oss+BMSC, and autogenous bone groups than in the empty control, MBCP-only, and Bio-Oss-only groups at week 2 and week 4. However, comparable results were observed among the MBCP-only, MBCP+DPSC, MBCP+BMSC, Bio-Oss-only, Bio-Oss+DPSC, and Bio-Oss+BMSC groups after 8 weeks of healing (Figure 6, Figure 7, Figure 8 and Figure 9).

After 2 weeks of healing, the connective tissue mainly filled the bone defect in the empty control group (Figure 6A). In the border of the defect, fibrous tissue formation was evident, with little woven bone formation along the margin of the defect. In the central area of the defect, the space was almost occupied by connective tissue. By contrast, the autogenous bone graft group exhibited the highest bone healing capacity, with more bone bridges and island formation in the whole bone defect (Figure 6H). In the MBCP-only and Bio-Oss-only groups, even in the central area of the defect, new bone formation was observed surrounding the bone grafting materials (Figure 6B,E). This phenomenon indicates that the bone grafting materials exhibited osteoconductive capabilities during the bone healing process. As shown in Figure 6B–D, comparable new bone formation was observed among the MBCP-only, MBCP+DPSC, and MBCP+BMSC groups. Close contact between the new bone and MBCP grafting materials and more new bone growth in the porous MBCP were observed after incorporating the DPSCs and BMSCs. Moreover, the Bio-Oss+DPSC and Bio-Oss+BMSC groups showed more new bone formation than the Bio-Oss-only group (Figure 6E–G).

After 4 weeks of healing, mild new bone formation was observed at the defect margin of the empty control group (Figure 7A). By contrast, increased new bone formation, increased bone thickness, and disappearance of the contact line between the autogenous bone graft and new bone were observed in the autogenous bone group (Figure 7H). In the MBCP- and Bio-Oss-only groups, moderate new bone formation was noted around the grafting materials (Figure 7B,E). Furthermore, the grafting material was gradually resorbed and replaced by the new bone after 4 weeks. The phenomenon of new bone growth in MBCP was more evident in the MBCP+DPSC and MBCP+BMSC groups than in the MBCP-only group (Figure 7C,D). Similar new bone formation and a remodeling tendency were observed in the Bio-Oss+DPSC and Bio-Oss+BMSC groups (Figure 7E,F). Moreover, an increased number of osteoblastic cells lining the surface of the newly formed bone and bone grafting materials were observed after incorporating the MSCs. These findings demonstrated that both the DPSCs and BMSCs enhanced the osteoconductive capacities of MBCP and Bio-Oss.

After 8 weeks of healing, moderate new bone formation was observed in the whole bone defect of the empty control group (Figure 8A). By contrast, the autogenous bone group presented a more compact and mature bone consisting of lamellar bone and marrow (Figure 8H). More MBCP and Bio-Oss grafting materials were resorbed and replaced by the new bone (Figure 8B,E). In addition, a more compact and mature lamellar bone was observed in the MBCP, MBCP+DPSC, MBCP+BMSC, Bio-Oss, Bio-Oss+DPSC, and Bio-Oss+BMSC groups when comparing the histological findings at 2 and 4 weeks (Figure 8C–G). However, no significant differences in the percentage of the compact lamellar bone were observed among these groups.

### 2.5. Histomorphometric Results

After 2 weeks of healing (Figure 9A), the percentage of the newly formed bone in the autogenous bone group (39.4% ± 4.7%) was significantly higher than that in other groups (*p* < 0.05), whereas the empty control group had a lower percentage of newly formed bone (14.9% ± 4.9%). However, no significant differences in the percentage of the newly formed bone were observed among the MBCP (27.2% ± 5.1%), MBCP+DPSC (28.3% ± 6.7%), MBCP+BMSC (30.1% ± 3.8%), Bio-Oss (24.7% ± 5.4%), Bio-Oss+DPSC (31.8% ± 3.7%), and Bio-Oss+BMSC (32.0% ± 2.8%) groups. After 4 weeks of healing (Figure 9B), the autogenous bone group showed the highest percentage of new bone formation (49.7% ± 1.5%; *p* < 0.05). Moreover, the Bio-Oss+BMSC (41.6% ± 5.4%) group had comparable results with those of the autogenous bone group. The empty control group showed the lowest percentage of new bone formation (24.7% ± 5.4%); however, their value did not differ from those of the MBCP (27.7% ± 7.5%) and Bio-Oss (32.6% ± 2.2%) groups. The results of the MBCP+DPSC (37.5% ± 3.3%) and MBCP+BMSC (38.5% ± 3.8%) groups were similar to that of the MBCP group, whereas the Bio-Oss+DPSC (39.2% ± 3.6%) and Bio-Oss+BMSC (41.6% ± 5.4%) groups showed a significantly higher percentage of new bone formation than the Bio-Oss group (32.6% ± 2.2%; *p* < 0.05). Finally, after 8 weeks of healing (Figure 9C), the autogenous bone group (54.9% ± 6.1%) showed the highest percentage of new bone formation compared to the empty control (35.3% ± 0.5%), MBCP (38.3% ± 6.0%), MBCP+DPSC (39.8% ± 5.7%), Bio-Oss (41.3% ± 3.5%), and Bio-Oss+DPSC (42.1% ± 2.7%) groups. Moreover, no significant differences were observed between the MBCP+BMSC (47.2% ± 8.3%) and Bio-Oss+BMSC (51.2% ± 9.9%) groups and the autogenous bone group. These results demonstrated that the efficiency of the BMSC groups combined with MBCP and Bio-Oss was comparable to that of the autogenous bone group.

## 3. Discussion

In tissue repair, cells or materials are directly transplanted to the injury site, and they eventually become part of the patient’s body [34,35,36]. However, the biological properties of transplanted materials and cells must be properly evaluated to ensure their successful integration with the host tissue and to prevent complications [37]. In the present study, we comprehensively analyzed the effects of MBCP and Bio-Oss scaffolds with or without DPSCs or BMSCs on the bone regeneration and repair of calvarial bone defects in rabbits and compared those results with those of the autogenous bone, which is the gold standard.

As shown in Figure 1, the SEM images showed that MBCP is a biphasic calcium phosphate synthetic bone grafting material with a unique micro- and macroporous structure [38]. MBCP can gradually dissolve and degrade in the body, and its porous structure becomes completely infiltrated with and replaced by newly formed bone. In addition, the release of calcium and phosphate ions can promote new bone formation. By contrast, Bio-Oss is a natural, nonantigenic, and porous bone mineral matrix produced by removing all organic components from the bovine bone. The inorganic bone matrix of Bio-Oss contains macroscopic and microscopic structures with an interconnecting pore system that serves as a biophysical scaffold for the immigration of osteogenic cells [39] (Figure 1). Due to its natural structure, Bio-Oss is physically and chemically comparable to the mineralized matrix and architecture of human bone [40]. The Bio-Oss particles become an integral part of the newly formed bone framework and preserve BV over the long term [41]. In clinical practice, both MBCP and Bio-Oss are recommended for dental applications as alternatives to autogenous bone because of their biocompatibility and higher osteoconductive potential [42]. Moreover, a comparative study showed that wider peaks in the XRD pattern of Bio-Oss indicated its less-crystalline nature compared with MBCP [1]. In terms of the enhancement of bone regeneration, MBCP can release more calcium and phosphate for promoting new bone formation, whereas Bio-Oss depends on the human bone-like structure for stimulating new bone regeneration. Moreover, the physicochemical properties of MBCP with micropores and microporosity are essential for osteoconduction, whereas Bio-Oss with the highest surface area and more amorphous structure induces stronger calcein and integrin signals to trigger bone formation [43]. Previous studies have also suggested that an ideal bone grafting material should possess features including an interconnected porosity with adequate pore size, surface structure, adequate mechanical properties, controlled biodegradability, sufficient dimensional stability, and the release of active bone-promoting biomolecules [5,6]. Moreover, achieving a balance between the resorption of bone grafting materials during the tissue remodeling process and the maintenance of the bone defect volume for new bone ingrowth will improve osteoinduction [6]. For example, Smartbone^®^, a xenohybrid bone graft, is commercially available as a CE-labeled class III medical device and has shown high levels of bioactivity when loaded with MSCs and lyosecretome [5,7].

In the present study, both the DPSCs and BMSCs had similarly fibroblastic and spindle-shaped morphology as MSCs. However, the BMSCs showed higher osteogenic and chondrogenic differentiation potential, whereas the DPSCs showed higher CFU and proliferation capacities (Figure 2). These results are consistent with those of previous in vitro studies [27,44,45,46]. Compared with BMSCs, DPSCs provide the advantages of harvesting cells from discarded teeth in a noninvasive manner, a high proliferation rate, a high odontogenic/osteogenic differentiation potential, and excellent immunomodulatory properties [31,47,48]. Simultaneously, the DPSCs exhibited a stronger antiapoptotic ability under the microenvironment of oxidative stress [45]. However, in vivo studies have reported controversial results for new bone formation by using DPSCs and BMSCs, and their efficiency in comparison with the autogenous bone was not examined in previous studies [27,44,47,49].

Our Micro-CT and histological analysis results indicated that the empty control group had the slowest healing ability (Figure 4, Figure 5, Figure 6, Figure 7, Figure 8 and Figure 9). The implantation of MBCP and Bio-Oss in bone defects provided osteoconduction capacities and scaffold support for enhancing new bone formation and preventing the collapse of the 3D healing space in the defect; however, their regenerative capacities are still far from those of the autogenous bone. Overall, no significant differences in the regenerative potential were observed between MBCP and Bio-Oss. However, the combination of MBCP and Bio-Oss with the DPSCs and BMSCs could exert positive and synergistic effects on bone regeneration. Although this phenomenon was not evident during the early healing period, comparable outcomes with the autogenous bone were noted after 8 weeks of healing (Figure 5 and Figure 9). Moreover, the DPSCs and BMSCs in combination with MBCP and Bio-Oss presented similar healing performances and tendencies in terms of bone regeneration. As shown in Figure 7, more osteoblast lining at the interface between MBCP/Bio-Oss and the newly formed bone was observed after the implantation with MSCs. In addition, a mouse study demonstrated that MBCP and Bio-Oss showed higher stem cell-carrying potentials [10]. However, the regenerative capacities of the MBCP+BMSC and Bio-Oss+BMSC groups were similar to those of the autogenous bone. On the basis of the aforementioned results, the BMSCs appeared to be more effective in enhancing bone regeneration when used in combination with MBCP and Bio-Oss compared with the DPSCs. However, the isolation of BMSCs from bone marrow is often painful and increases the risk of infection. Thus, while selecting an adequate MSC source, practitioners should consider the advantages and disadvantages of specific MSCs according to the clinical requirements.

The regeneration process of MSCs involves two potential mechanisms: direct proliferation or differentiation and indirect paracrine function [50,51]. MSCs can proliferate as undifferentiated stem cells and differentiate into various lineages, depending on the microenvironment, with the replacement of loose endogenous cells and damaged tissues [51]. Moreover, the secretion of bioactive soluble factors and extracellular vesicles (including exosomes and microRNAs) through their paracrine effects of MSCs support the survival of endogenous cells and the improvement of the microenvironment by modulating the angiogenesis, osteogenesis, and immune responses; suppressing apoptosis; reducing oxidative stress; and recruiting tissue-specific progenitor cells [52,53,54]. These direct and indirect mechanisms may work individually or synergistically. After implantation in bone defects, the biomaterials combined with MSCs and the regenerated bone matrix formed a complex network resembling the endogenous bone structure, which is critical to facilitate new bone formation and create an osteoinduction-like environment or niche to improve the bone healing efficiency of these xenografts and synthetic bone grafts.

Some limitations still exist for obtaining an adequate number of MSCs to meet the clinical dose requirements, including a low harvesting quantity and the degradation and aging of MSCs following long-term culturing [55]. Moreover, extrinsic factors such as the health status of the donor, aging, and a low oxygen level can significantly and negatively affect the efficiency of clinical cell transplantation [49,55]. Although overcoming extensive bone loss and improving the therapeutic efficacy remain challenging, novel MSC-based bone regeneration strategies, including cytotherapy, 3D culture, preconditioning, and cell-free approaches, appear promising for curing severe large bone defects, irrespective of the regular or diseased microenvironments [15,27,56].

## 4. Materials and Methods

### 4.1. Characterization of MBCP and Bio-Oss Bone Grafting Materials

MBCP (Biomatlante, Vigneux de Bretagne, France) is a nonstructural bone substitute with biological activity, and it consists of a homogenous distribution of a 60% hydroxyapatite (HA) and 40% β-tricalcium phosphate crystalline structure. The overall porosity and size of MBCP are 70% and from 0.5 to 1 mm, respectively. Bio-Oss (0.25–1 mm, Geistlich Pharma AG, Wolhusen, Switzerland) is a bovine-derived xenograft.

The surface morphologies of MBCP and Bio-Oss were evaluated using a scanning electron microscope (SEM). In brief, MBCP and Bio-Oss were pasted on a conductive tape, coated with a thin layer of gold by using a sputter coating machine, and observed using an SEM (SU3500, Hitachi High-Technologies Corporation, Tokyo, Japan) with an accelerating voltage of 15 kV.

### 4.2. Isolation and Culture of DPSCs and BMSCs

The DPSCs were isolated from freshly extracted incisors by using the direct outgrowth method described previously [22,27]. The pulp tissue was carefully removed from each tooth by using a sterile mortar and pestle. The tissue was washed with phosphate-buffered saline (PBS) three times, minced into pieces, and then cultured in 3.5-cm-diameter Petri dishes at 37 °C in a 5% CO_2_ environment. The DPSCs were cultured in alpha minimum essential medium (α-MEM, Gibco/Invitrogen, Carlsbad, CA, USA) containing 10% fetal bovine serum (Gibco/Invitrogen), 1% antibiotic–antimycotic solution (Sigma-Aldrich, St. Louis, MO, USA), 10 mL of antibiotic–antimycotic solution (Sigma-Aldrich), and 0.5% L-ascorbic acid 2-phosphate. DPSCs were passaged through a detachment with 0.5% trypsin–EDTA solution when the cell culture reached ≥80% confluence. The DPSCs were passed through a 70-µm strainer (BD Falcon, San Jose, CA, USA). Subsequently, isolated DPSCs were collected based on their small size. Harvested DPSCs were cultured in 10-cm-diameter Petri dishes for future investigation.

To isolate the BMSCs, approximately 1 mL of the bone marrow was harvested through needle aspiration from the tibial or femoral bones of anesthetized rabbits and suspended in 2 mL of PBS, as described previously [34]. The bone marrow suspension was layered on 3 mL of Ficoll–Hypaque Plus solution (GE Healthcare BioSciences Corp., Piscataway, NJ, USA) for density gradient centrifugation at 400× *g* for 30 min. The mononuclear cell layer was collected and washed twice with α-MEM. Subsequently, isolated mononuclear cells were cultured in α-MEM containing 1% antibiotic–antimycotic solution (Sigma-Aldrich, St. Louis, MO, USA), 10 mL of antibiotic–antimycotic solution (Sigma-Aldrich), and 0.5% L-ascorbic acid 2-phosphate at 37 °C in a humidified atmosphere of 95% air and 5% CO_2_. After 5 days of culture, nonadherent cells were rinsed away, and fresh medium was added. The culture medium was changed at day 5 to remove nonadherent cells and exchanged every 3 days. The culture medium was then changed every 2 to 3 days. When the cells reached 80–90% confluence, they were subcultured in α-MEM. All cells were passaged through a detachment with trypsin when the culture reached ≥70% confluence. Unsorted or otherwise enriched DPSCs and BMSCs were cultured.

Before using the DPSCs and BMSCs in further experiments, their multilineage differentiation capacity was confirmed in osteogenic and chondrogenic induction media. In addition, the colony-forming unit (CFU) efficiency of the DPSCs and BMSCs was examined. Cells at passages 3–6 were used in experiments to ensure the retention of their stem cell qualities. Furthermore, cells at passages 2–8 were used in subsequent in vitro experiments to ensure the retention of their stem cell qualities.

### 4.3. Animals and Ethics

All animal experimental procedures were performed in compliance with the guidelines of and after obtaining ethical approval from the Institutional Animal Care and Use Committee of Taipei Medical University, Taipei, Taiwan (approval no. LAC-2017-0126) under the ARRIVE guidelines [35]. In total, 24 adult male New Zealand white rabbits weighing between 3.5 and 4.0 kg were used in this study; four 6-mm-diameter calvarial defects were created in these rabbits. The animals were individually housed in the Central Animal Facility at Taipei Medical University under standard environmental conditions (temperature: 22 °C ± 2 °C, humidity: 30–60% ± 5%, and a 12/12-h light/dark cycle) with ad libitum access to food and drinking water.

### 4.4. Animal Experiments and Surgical Procedures

The animals were anesthetized through an intramuscular injection of tiletamine–zolazepam at a dose of 15 mg/kg (Zoletil 50, Virbac, Carros Cedex, France) and xylazine at a dose of 5 mg/kg. After the application of local anesthesia with 1.8 mL of 2% lidocaine (1:100,000 epinephrine) and disinfection of the surgical site with beta iodine, a midline skin incision was made, followed by muscle dissection and periosteal elevation. The calvarial bone was exposed, and a 6-mm-diameter trephine drill was used to create four circular calvarial bicortical bone defects under the copious irrigation of sterile saline (Figure 3a). Care was taken to prevent injury of the dura. The following eight treatment modalities were randomly allocated to bone defects: (1) empty control, (2) MBCP, (3) MBCP+DPSCs, (4) MBCP+BMSCs, (5) Bio-Oss, (6) Bio-Oss+DPSCs, (7) Bio-Oss+BMSCs, and (8) autogenous bone. The DPSCs and BMSCs (1.0 × 106) in 0.5 mL of PBS were homogeneously mixed with MBCP and Bio-Oss (30 mg) before implantation in bone defects. After surgery, the muscle layer was closed using a bioresorbable suture (Vicryl 4.0, Ethicon, Somerville, NJ, USA), and the skin layer was sutured using a nylon suture. Antibiotics (Baytril, Bayer, Leverkusen, Germany) (5.0 mg/kg, SC, BID) and analgesics (Rimadyl, Pfizer, New York, NY, USA) (4.0 mg/kg, SC, BID) were administered postoperatively for 3 days to prevent wound infection and relieve pain. The surgical wounds, food intake, and activity of the animals were monitored daily.

### 4.5. Micro-Computed Tomography Measurements

To examine the new bone formation, after 2, 4, and 8 weeks of healing, the rabbits were sacrificed, and their tissue blocks were harvested. After fixation in 10% neutral-buffered formalin for 3 days, the samples were processed and scanned using micro-computed tomography (micro-CT) equipment (Bruker Skyscan 1172, Bruker, Kontich, Belgium) at a voltage of 50 kV, an electric current of 100 mA, and a pixel resolution of 18 µm with a 0.5-mm aluminum filter. Subsequently, reconstructed three-dimensional (3D) image models were imported into the analysis software (CTAn, Bruker, Billerica, MA, USA) to calculate the bone volume (BV). The optimal thresholds were set for segmenting the micro-CT images to differentiate the newly formed bone from the connective tissue and grafting materials. Finally, the percentage of BV to the total tissue volume (TV) (BV/TV%) within the volume of interest (VOI) was evaluated and expressed as the mean ± standard deviation.

### 4.6. Histology and Histomorphometric Analyses

After the micro-CT measurements, the harvested samples were prepared for the histological and histomorphometric analyses. The samples were decalcified in Plank-Rychlo’s solution (MUTO Pure Chemicals Co., Tokyo, Japan) for 5 days, dehydrated in graded ethanol concentrations, and then embedded in paraffin. The embedded samples were longitudinally cut into 4-µm-thick sections and stained with hematoxylin and eosin (H&E; Sigma, St. Louis, MO, USA). For the qualitative analysis of the bone regenerative process, the stained samples were evaluated, especially in the border and center areas, using a standard light microscope (Leica DM500, Leica Microsystems, Wetzlar, Germany) connected to a SPOT digital camera (Diagnostic Instruments, Inc., Sterling Heights, MI, USA). Four sites in each sample were randomly selected to calculate the new bone formation percentage by using Image-Pro Plus 6.0 software (Media Cybernetics, Silver Spring, MD, USA).

### 4.7. Statistical Analysis

The statistical analyses were performed using SPSS for Windows (Version 19, SPSS Inc., Chicago, IL, USA). The results were presented as the mean ± SD. The differences among all the experimental groups were examined using a one-way analysis of variance (SPSS Inc., Chicago, IL, USA), followed by Tukey’s honest significant difference test. The data were considered to be significantly different if the *p*-values were <0.05.

## 5. Conclusions

In summary, our Micro-CT and histological findings demonstrated that the autogenous bone is the gold standard for bone regeneration in a rabbit calvarial bone defect model. Moreover, the space maintenance ability of bone grafting materials and the bioactivity of MSCs can synergistically enhance new bone formation. Furthermore, this effective and clinically translatable approach will eventually be referring to the major world systems (US-FDA, EU, China, etc.).

## Data Availability

Not applicable.
